# The Tmem16a chloride channel is required for mucin maturation after secretion from goblet-like cells in the *Xenopus tropicalis* tadpole skin

**DOI:** 10.1038/s41598-024-76482-y

**Published:** 2024-10-26

**Authors:** Eamon Dubaissi, Emma N. Hilton, Sarah Lilley, Richard Collins, Charlotte Holt, Peter March, Henry Danahay, Martin Gosling, Richard K Grencis, Ian S Roberts, David J Thornton

**Affiliations:** 1 School of Biological Sciences, University of Manchester, Manchester, M13 9PT UK; 2 Lydia Becker Institute of Immunology and Inflammation, University of Manchester, Manchester, M13 9PT UK; 3 Wellcome Centre for Cell Matrix Research, University of Manchester, Manchester, M13 9PT UK; 4 Faculty of Biology, Medicine and Health, University of Manchester, Manchester, M13 9PT UK; 5 Sussex Drug Discovery Centre, University of Sussex, Falmer, Brighton, BN1 9QJ UK; 6 Enterprise Therapeutics, Sussex Innovation Centre, Science Park Square, Falmer, Brighton, BN1 9SB UK

**Keywords:** Mucin, Mucus, TMEM16A, Ion channel, *Xenopus tropicalis*, Developmental biology, Disease model, Immunology, Mucosal immunology, Ion channels, Biochemistry, Electron microscopy

## Abstract

The TMEM16A chloride channel is proposed as a therapeutic target in cystic fibrosis, where activation of this ion channel might restore airway surface hydration and mitigate respiratory symptoms. While TMEM16A is associated with increased mucin production under stimulated or pro-inflammatory conditions, its role in baseline mucin production, secretion and/or maturation is less well understood. Here, we use the Xenopus tadpole skin mucociliary surface as a model of human upper airway epithelium to study Tmem16a function in mucus production. We found that *Xenopus tropicalis* Tmem16a is present at the apical membrane surface of tadpole skin small secretory cells that express canonical markers of mammalian “goblet cells” such as Foxa1 and *spdef. X. tropicalis* Tmem16a functions as a voltage-gated, calcium-activated chloride channel when transfected into mammalian cells in culture. Depletion of Tmem16a from the tadpole skin results in dysregulated mucin maturation post-secretion, with secreted mucins having a disrupted molecular size distribution and altered morphology assessed by sucrose gradient centrifugation and electron microscopy, respectively. Our results show that in the Xenopus tadpole skin, Tmem16a is necessary for normal mucus barrier formation and demonstrate the utility of this model system to discover new biology relevant to human mucosal biology in health and disease.

**Supplementary Information:**

The online version contains supplementary material available at 10.1038/s41598-024-76482-y.

## Introduction

Aberrant mucin production is a hallmark of airway diseases including asthma, cystic fibrosis (CF) and chronic obstructive pulmonary disease^1^. Excessive mucus production and/or dehydrated mucus can cause airway blockages, defective mucociliary clearance (MCC) and vulnerability to infection^2,3^. Mucins, the major structural component of mucus, are large, polymeric glycoproteins that require water for remodelling and expansion upon secretion from epithelial secretory cells and submucosal glands^4^. Understanding how mucins are packaged in secretory granules and, post-secretion, unfold to form the hydrated mucus network is critical for the development of therapeutics to tackle obstructive lung disease. In CF, the cystic fibrosis transmembrane conductance regulator (CFTR) ion channel is dysfunctional^5–7^. Ionic imbalance leads to a dehydrated airway surface and mucus hyper-concentration^8^, and mucins that cannot properly expand^9^. This results in a more viscoelastic mucus not effectively cleared by cilia and chronic colonisation of mucus by potentially pathogenic organisms^10,11^. This devastating disease highlights the importance of ionic homeostasis for the maintenance of a healthy mucus barrier at the airway surface. Recently, several effective therapeutics have been developed to treat CF by directly modulating CFTR^12,13^. However, not all CFTR mutations are tractable to these drugs, and an unmet medical need within the genetically-diverse patient populations remains^14^. Accordingly, mutation-agnostic approaches have been developed to target other ion channels present in the airway, aiming to restore hydration of the airway surface.

One candidate channel is the calcium-activated chloride channel TMEM16A (ANO1; in non-humans, Tmem16a/Ano1)^15–17^. TMEM16A is present in many different cell types and associated with multiple physiological roles, including epithelial secretion^18–20^. In healthy airway epithelium, it is principally present in mucin-secreting goblet cells (GCs)^21^. TMEM16A levels increase following GC hyperplasia in asthmatic airways^22–24^and when mice are subjected to inflammatory cytokines^25^. The TMEM16A channel is activated by calcium^26–28^, voltage dependent^29,30^ and can transport both chloride and bicarbonate ions^31,32^. It is a candidate for treatment of CF airway disease because movement of chloride/bicarbonate through TMEM16A, rather than CFTR, could restore hydration of the airways^15,33^. Indeed, TMEM16A potentiation can increase airway surface liquid (ASL) volume and restore MCC in an ovine model of CF^16^.

Evidence for TMEM16A function in healthy mucus production in unchallenged airway epithelia is conflicting^34,35^. Studies of TMEM16A function in healthy airways has largely been conducted in ex vivo tissue or in vitro airway epithelial cells. in vivo studies are complicated by the anatomical location of the airways deep within the body of mammalian model organisms. In recent years, the Xenopus tadpole skin has been used to study mucociliary epithelia. It has motile multiciliated cells, mucin-secreting cells and ionocytes^36,37^, and recapitulates the structure of the mammalian upper airway epithelium but with the advantage of being exposed to the environment and thus, easily visualised and manipulated. The gel-forming mucin MucXS, secreted by the tadpole skin, has properties similar to human airway mucins - it is large, polymeric and heavily glycosylated, and generates a barrier that can trap bacteria^38^. Here, in *Xenopus tropicalis*, we find that Tmem16a is expressed in the tadpole skin, at the apical membrane of a secretory cell type that resembles human GCs. We show that *X*. *tropicalis* Tmem16a is functionally equivalent to human TMEM16A in its sensitivity to calcium and voltage, and also its response to specific inhibitors. Further, depletion of Tmem16a affects the amount and macromolecular properties of MucXS secreted in response to the secretagogue, ionomycin. Our data demonstrate the utility of the *X*. *tropicalis* tadpole as a model to study mucus biology, revealing that Tmem16a can influence gel-forming mucin properties after secretion, and hence may modulate mucus barrier structure.

## Results

### *X. tropicalis tmem16a* is expressed in mucin-producing small secretory cells in tadpole skin

Functional studies of Tmem16a in Xenopus are limited to its role in the oocyte and the prevention of polyspermy^39–41^. RT-PCR expression analysis in adults shows expression in various tissues^42,43^, while bulk RNA sequencing in developing embryos shows expression at Nieuwkoop-Faber (NF) stages NF1, NF9 and NF24-NF42^42^. We therefore sought tissue-specific expression during skin development.

**Fig. 1:**
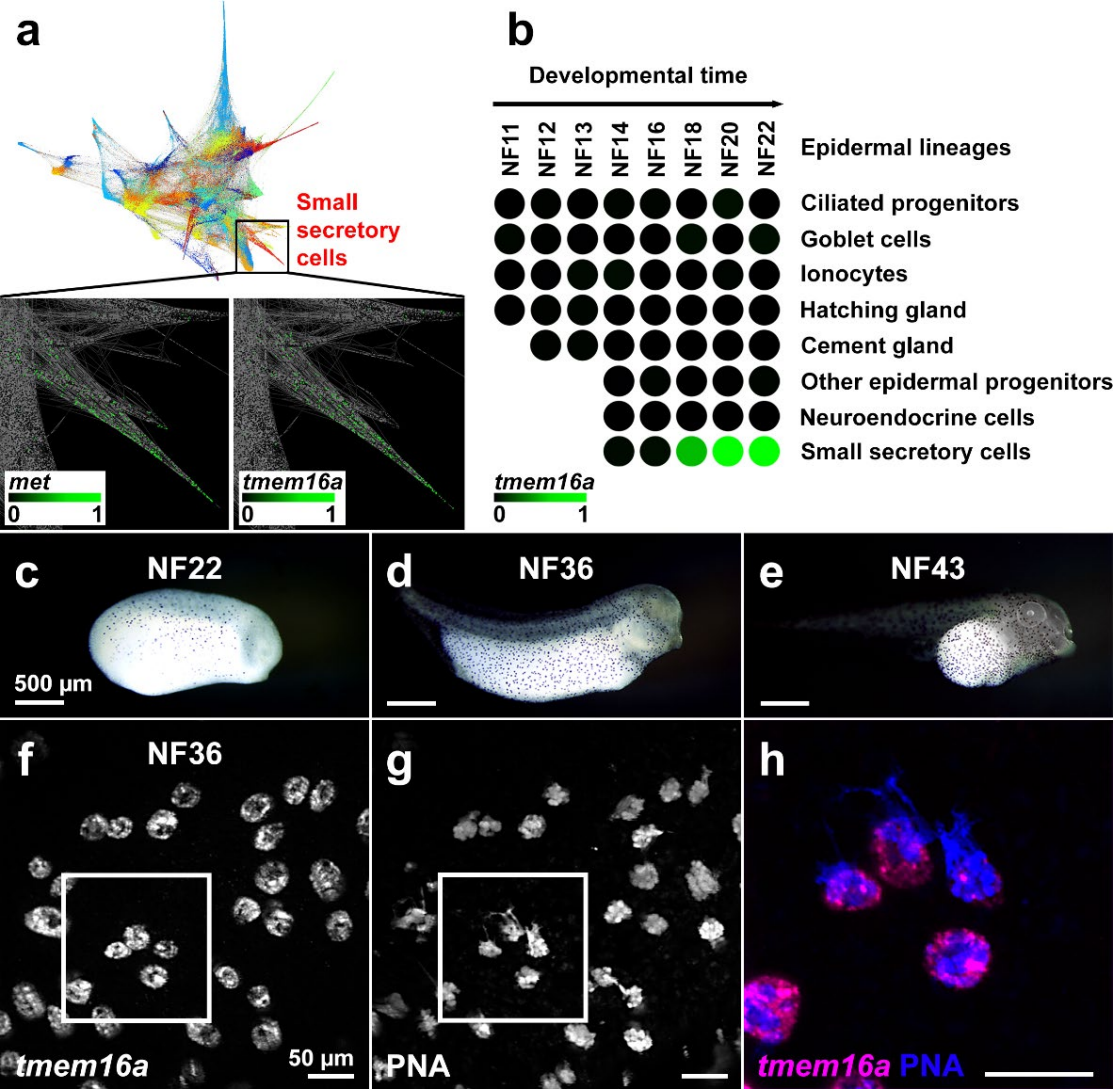
*X. tropicalis tmem16a* is expressed in SSCs in the tadpole skin. (**a**–**b**) In a developmental time series of single-cell transcriptomes in *X*. *tropicalis* embryos from NF8-NF22, an SSC lineage (highlighted in red) was identified (**a**, upper). An expanded view (black box) examining gene-specific expression in this SSC lineage reveals expression of *met* (**a**, lower left; marking this SSC lineage) and of *tmem16a* (**a**, lower right). Within epidermal lineages, *tmem16a* expression is present in the SSC lineage from NF18 but absent from other epidermal lineages from differentiation to NF22 (**b**). Each circle represents a developmental stage in a specific epidermal lineage. Scale bars: colour intensity represents normalised expression level from undetectable (0) to maximum expression (1) within the entire dataset. (**c**–**e**) Chromogenic in situ hybridisation for *tmem16a* at stages NF22 (**c**), NF36 (**d**) and NF43 (**e**) reveals punctate expression throughout the skin, matching typical SSC distribution. (**f**–**h**) Fluorescent in situ hybridisation for *tmem16a* (**f**) and dual staining with the SSC vesicle marker PNA (**g**) confirms that *tmem16a* is expressed in SSCs (merged and expanded from white box in **h**).

Briggs^43^ generated a developmental time series of single-cell transcriptomes in *X*. *tropicalis* embryos, covering early development from pre-gastrulation (NF8) to early tailbud (NF22), and disaggregated multiple cell lineages. Online interrogation of this dataset^44^ permits analysis of gene expression in specific cell types. Visualised as an ‘all stages’ SPRING plot^45^, the mucin-producing small secretory cell (SSC) lineage (Fig. 1a, upper) was originally identified by expression of *met* (Fig. 1a, lower left)^43^. *tmem16a* is expressed within this *met*-marked SSC lineage (Fig. 1a, lower right). No other clusters of expression in other cell lineages between NF8-NF22 were observed in this ‘all stages’ SPRING plot. To confirm specific expression in the SSC lineage and not other epidermal lineages, we analysed epidermal lineages in ‘tree view’. Over developmental time, the SSC lineage differentiates at NF14 from a pool of non-neural ectodermal cells that, from NF11 onwards, give rise to the major cell lineages of the tadpole skin. Analysis of *tmem16a* expression over differentiation of these epidermal lineages revealed expression only in SSCs in the developing epidermis, from NF18 and increasing to NF22 (Fig. 1b).

Using chromogenic mRNA in situ hybridisation, we tested *tmem16a* expression over a later time course of tadpole development (NF22, NF36 and NF43). We identified *tmem16a* expression in a punctate pattern in the tadpole skin at these later stages, matching our published distribution of SSCs (Fig. 1c–e)^46^. To confirm *tmem16a* expression is in SSCs at these later stages, we performed fluorescent in situ hybridization and staining with peanut agglutinin lectin (PNA), a lectin that binds to the Gal(β1–3)GalNAc moieties of mucin O-glycans and robustly marks the mucin-containing vesicles of SSCs^46^. Confocal imaging colocalised *tmem16a* with PNA in SSCs (Fig. 1f–h).

The above expression analyses show that *X*. *tropicalis tmem16a* is expressed exclusively in SSCs in the tadpole skin. In support, a single-cell transcriptome dataset analysing lineage patterns in the related species *Xenopus laevis* found that *tmem16a* is detectable only in the SSC lineage of the developing skin^47^. As SSCs produce the gel-forming mucin MucXS^38^, these data suggest that *X*. *tropicalis* Tmem16a protein likely has a developmental and/or functional role in the mucociliary epidermal surface of the Xenopus tadpole.

### The mucin-producing SSCs are equivalent to mammalian goblet cells

We have shown that *tmem16a* expression is apparently exclusive to the SSCs in the developing *X*. *tropicalis* tadpole skin. However, in addition to SSCs, the *X. tropicalis* epidermis has another mucin-producing cell type termed goblet cells (GCs)^48^, implying equivalence with mammalian airway GCs that typically express *TMEM16A* and the mucin *MUC5AC*^49^. Thus, the expression pattern for *X*. *tropicalis tmem16a* generates a question regarding the homology of cell types in the Xenopus skin with those in the mammalian airway.

**Fig. 2:**
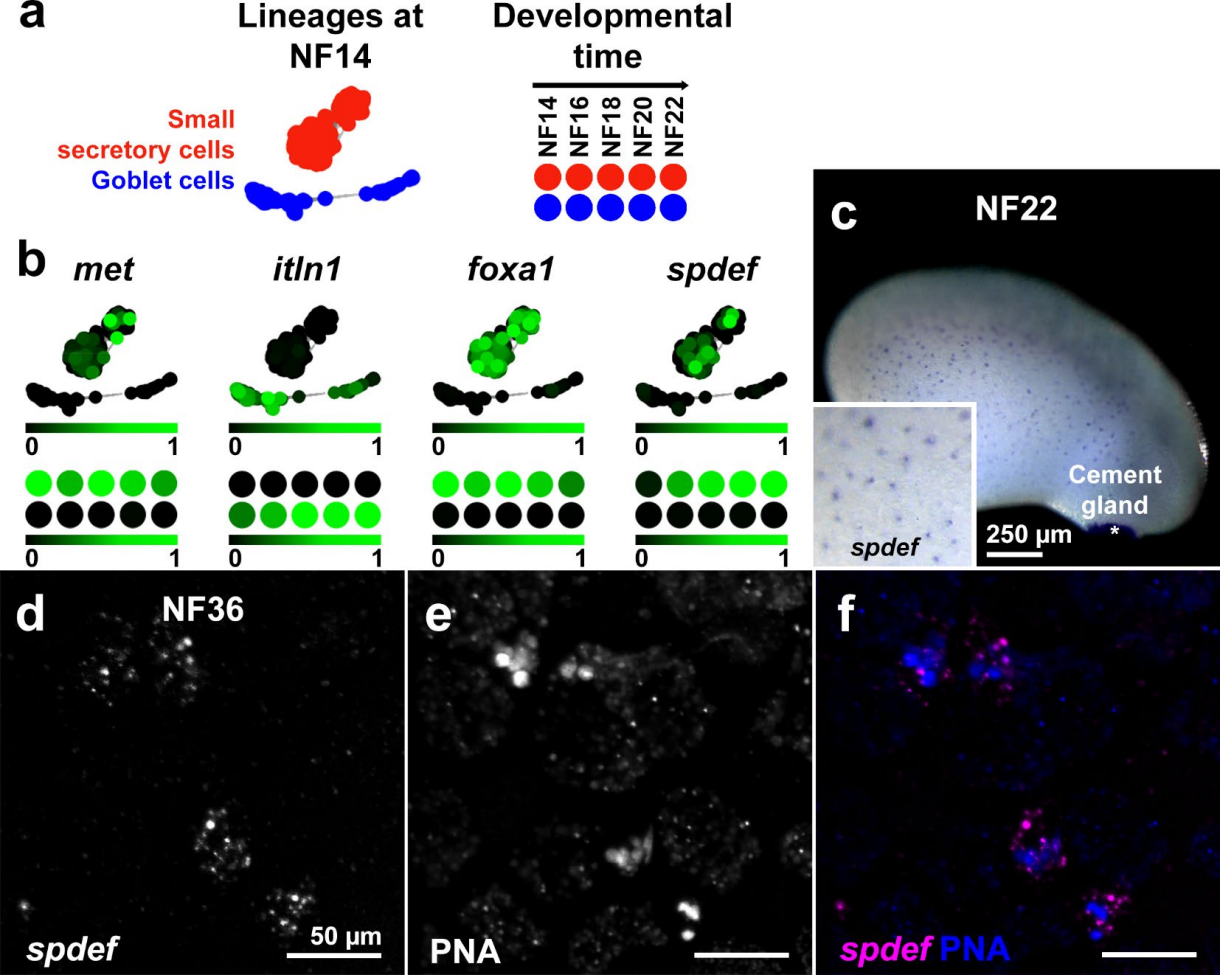
SSCs are a discrete epidermal cell type that express classical airway GC markers. (**a**–**b**) SSC (red) and GC (blue) lineage clusters in *X*. *tropicalis* embryos at NF14 and from NF14-22, are discrete (**a**) and marked by lineage-specific expression of *met* and *itln1*, respectively (**b**). The canonical goblet cell markers, *foxa1* and *spdef*, are expressed almost exclusively in the SSC cell cluster at NF14, and over developmental time (**b**). Scale bars: colour intensity represents normalised expression level from undetectable (0) to maximum (1) within the dataset. **c**. Chromogenic in situ hybridisation for *spdef* at NF22 reveals punctate expression throughout the skin (inset), typical of SSC distribution, and strong expression in the cement gland (asterisk). (**d**–**f**) Fluorescent in situ hybridisation of *spdef* mRNA (**d**) and dual staining with PNA (**e**) confirms that *spdef* is expressed in SSCs (merged in **f**).

Returning to the single-cell developmental transcriptome dataset, we captured SSC and GC lineages at NF14 (the developmental time at which SSCs differentiate from non-neural ectoderm) and, using the platform tools with default parameters, plotted the two discrete gene expression clusters (Fig. 2a, left). We also captured SSC- and GC-specific lineages over developmental time (Fig. 2a, right). The SSC and GC lineages are marked by expression of *met* and *itln1*, respectively, with lineage-specific expression evident in NF14 clusters and from NF14 to NF22 (Fig. 2b). We and others have previously shown that the canonical goblet cell marker *foxa1*is expressed in SSCs from NF14 and is necessary for the development of this cell population^46,50^. Within the transcriptome dataset, *foxa1* expression was near-exclusive to the SSC lineage at NF14 and from NF14 to NF22 (Fig. 2b).

We then sought expression of the transcription factor *spdef*, another mammalian GC marker^51^. We identified *spdef* expression in the NF14 SSC cluster and increasing levels of expression in SSCs from NF14 to NF22, but not in GCs at any stage analysed (Fig. 2b). In cultured human airway cells, FOXA1 has been shown to directly regulate transcription of *SPDEF*^52^. Here, we find that *X*. *tropicalis* SSCs express *foxa1* earlier (at NF14) than *spdef* (from NF16), suggesting the same transcriptional dynamic in tadpole skin SSCs.

To confirm *spdef* expression in SSCs in the developing tadpole skin, we used chromogenic in situ hybridisation in NF22 embryos and found punctate expression in the skin, typical of SSC distribution, and strong expression of the *spdef* gene in the cement gland, a mucin-producing organ located in the anterior region of the developing head (Fig. 2c and inset). Fluorescent in situ hybridisation in combination with PNA staining at NF36 localised *spdef* expression to SSCs containing PNA-positive secretory vesicles (Fig. 2d-f).

These data show early differentiation of the two known secretory cells types in the *X*. *tropicalis* skin. Further, it is the SSCs and not the skin cells termed GCs that express markers more typical of mammalian airway GCs. Thus, *tmem16a* expression in the *X*. *tropicalis* skin is in the cell type (SSCs) likely to replicate the biology of mammalian GCs.

### *X. tropicalis* Tmem16a protein localises to the plasma membrane of mucin-producing SSCs

TMEM16A has been localised to the apical plasma membrane in the mammalian airway epithelium^53^ in GCs secreting MUC5AC^54^, particularly during inflammation^55^. However, roles at the basolateral compartment of the plasma membrane in intestinal cells have also been described, as has the intracellular location of other paralogues of the TMEM16 family^56,57^. We hypothesised that a functional role in mucin secretion/processing in the tadpole skin would most likely arise from apical expression of Tmem16a.

**Fig. 3:**
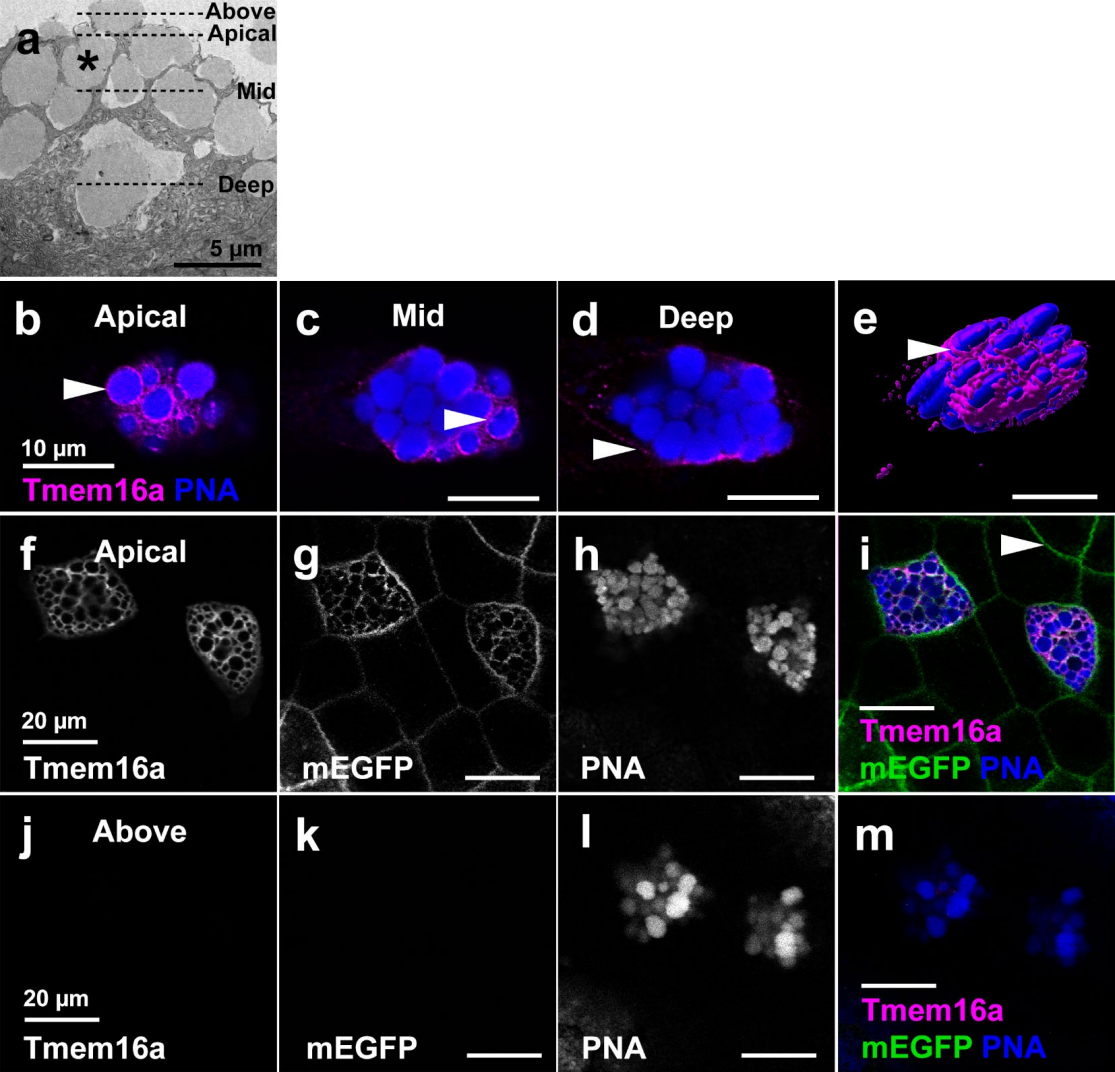
Tmem16a localises to the plasma and not vesicle membrane in SSCs. (**a**) SEM of SSCs reveals the presence of large vesicles (example indicated by asterisk) that bulge beyond the apical cell membrane of SSCs. Dashed lines indicate the planes of view in (**b**–**m**). (**b**–**e**) Immunofluorescent localisation of Tmem16a protein in apical, mid and basal planes of a single SSC reveals Tmem16a is present in a pattern that, in the apical (**b**) and mid (**c**) planes, appears to surround the PNA-positive secretory vesicles regions of cell (arrowheads). However, this localisation pattern is absent around vesicles in the more basal region of the cell (**d**), and expression becomes evident in the presumed plasma membrane (**d**, arrowhead). 3D surface rendering shows Tmem16a at an apical plane below that of the bulging vesicles (**e**). (**f-i**) In the apical plane of epidermal cells, Tmem16a (**f**) localises with mEGFP (g) in SSCs stained with PNA (**h**; merged in **i**), but does not localise with mEGFP marking the plasma membrane of other cell types (**h**, arrowhead). (**j**–**m**) Above the plane of epidermal cells, Tmem16a (**j**) and mEGFP (**k**) immunofluorescence is absent from bulging PNA-positive secretory vesicles (**l**; merged in **m**), demonstrating that Tmem16a is absent from the secretory vesicle membrane.

We investigated the cellular localisation of *X*. *tropicalis* Tmem16a protein in different planes of individual SSCs in the tadpole skin at NF36 by confocal microscopy, using an antibody against the human TMEM16A protein. The SSCs are filled with large, mucin-containing vesicles, visible by scanning electron microscopy (SEM) as bulges at the apical surface of the cell (Fig. 3a). At the SSC apical surface, Tmem16a appeared to surround the PNA-positive vesicles (Fig. 3b, arrowhead), also observable in the mid-plane of the cell (Fig. 3c). However, deep into the cell, expression was absent from the vesicle boundaries and appeared adjacent to the PNA-positive vesicles (Fig. 3d). Given the typical bulging of vesicles from the apical surface, we hypothesised that Tmem16a is, in fact, apical plasma membrane expression disrupted by this vesicle bulging, and this is supported by Tmem16a 3D surface rendering(Figure 3e).

To confirm plasma membrane localisation, we overexpressed membrane-localising EGFP (mEGFP) mRNA, targeting the ventral blastomeres fated to develop into skin to optimise signal. Tmem16a colocalised with mEGFP at the apical plasma membrane of PNA-positive SSCs (Fig. 3f–i), but not with mEGFP at the plasma membrane of other epidermal cell types (for example, Fig. 3i, arrowhead). Neither Tmem16a nor mEGFP signal was evident when imaging above the apical surface of the skin, while PNA-positive vesicles were evident in this plane (Fig. 3j–m).

We conclude that Tmem16a is found in the SSCs in the apical plasma membranes, and not the boundaries of mucin-containing vesicles. Thus, cellular localisation of Tmem16a is appropriate for a functional role in mucin secretion/processing.

### *X. tropicalis* Tmem16a has calcium-activated, voltage-dependent chloride channel activity

Previous studies have shown that *X*. *laevis* Tmem16a is a calcium-activated chloride channel that is voltage-dependent^26^and sensitive to inhibitors^41^. However, the pharmacology of *X*. *tropicalis* Tmem16a has not been characterised, and these data are relevant to understand the potential of the tadpole skin as a model for TMEM16A in human health.

**Fig. 4:**
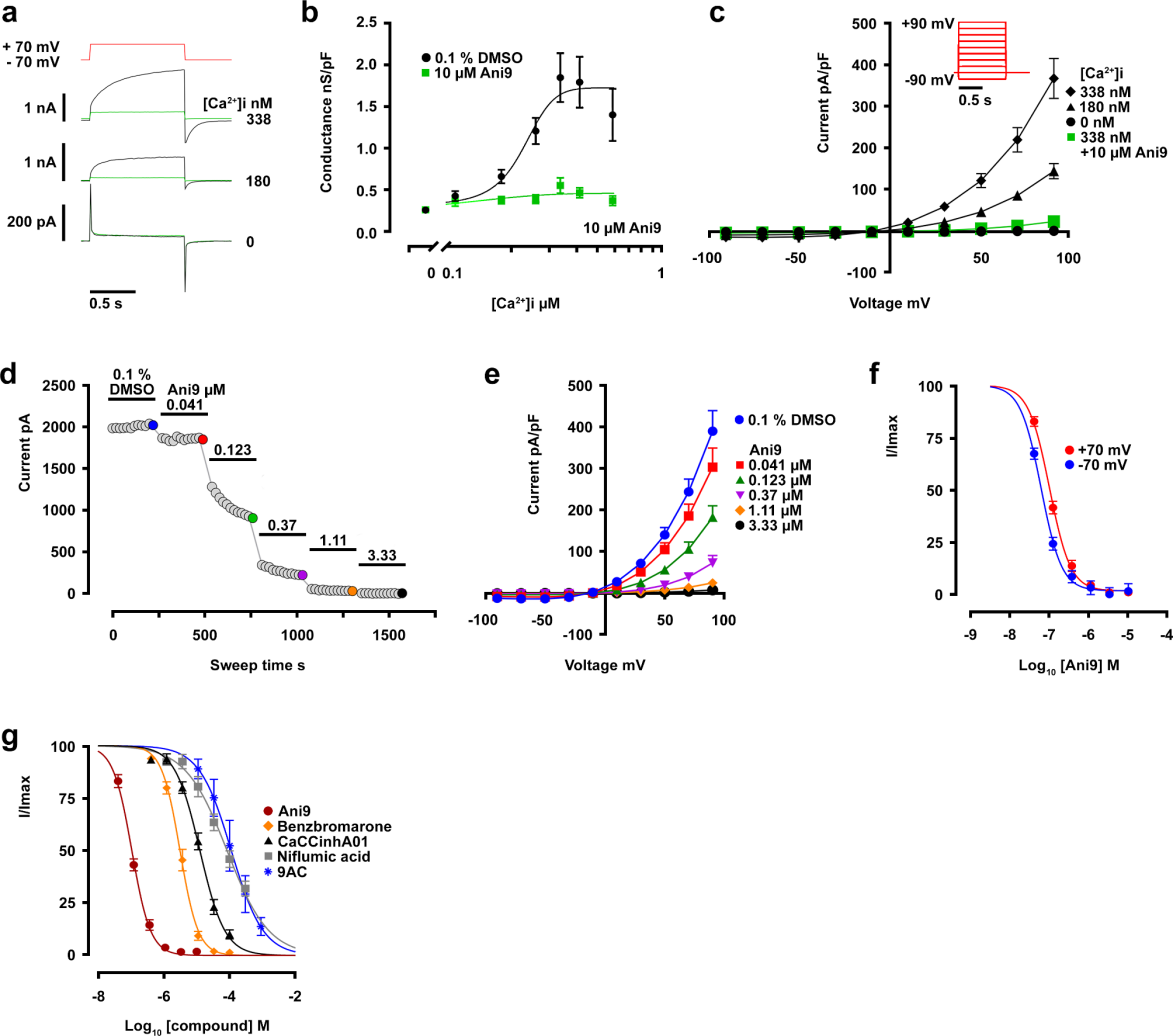
Biophysical and pharmacological characterisation of *X. tropicalis* Tmem16a. (**a**) Example currents evoked by single depolarizing pulses from − 70 to + 70 mV when [Ca^2+^]_i_ is 0, 180 (Ca^2+^ EC_20_) or 338 nM (Ca^2+^ EC_100_), with inhibition in the presence of 10 μm Ani9 shown in green. (**b**) *X*. *tropicalis* Tmem16a conductance is dependent on intracellular calcium levels (black) and inhibited in the presence of Ani9 (green). (**c**) In whole-cell current-voltage (I-V) tests recorded at 0, 180 & 338 nM [Ca^2+^]_i_, the inhibitory effect of Ani9 on maximally-active current (at 338 nM [Ca^2+^]_i_) across the voltage range is shown in green. (**d**–**f**) Further exploration of *X*. *tropicalis* Tmem16a sensitivity to Ani9, conducted at 338 nM (EC_100_) intracellular [Ca^2+^], finds concentration-dependent current inhibition recorded from a single cell at + 70 mV (**d**). Increasing concentration of Ani9 elicits dose-dependent inhibition of *X*. *tropicalis* Tmem16a (**e**). Both outward and inward chloride movement (recorded at -70 mV and + 70 mV respectively) is sensitive to inhibition by Ani9 (**f**). (**g**) *X*. *tropicalis* Tmem16a currents are sensitive to a range of frequently-used inhibitor compounds. All compounds were tested on maximally-active *X*. *tropicalis* Tmem16a currents (EC_100_ [Ca^2+^]_i_, + 70 mV).

We cloned and expressed full-length *X*. *tropicalis tmem16a* in HEK293 cells. At 48 h post-transfection, channel conductance and pharmacological characteristics were examined by whole-cell voltage clamp. Tmem16a channel activity was evoked using membrane depolarisation in the presence of intracellular calcium, and resultant whole-cell currents measured using chloride-selective buffers. A 1 s-step depolarisation from − 70 to + 70 mV in the presence of 338 nM free [Ca^2+^]_i_ evoked a large current (2.15 ± 0.51 nA, *n* = 18), which was slow to activate (tau_act_ 146 ± 34 ms, *n* = 18) and deactivate (tau_deact_ 90 ± 21 ms, *n* = 18), and was completely inhibited by the presence of 10 µM Ani9 (Fig. 4a)^58^. These activation kinetics and Ani9 sensitivity are key similarities that *X*. *tropicalis* Tmem16a shares with human TMEM16A, which distinguish both from the closest human homologue TMEM16B^59^.

Varying the level of free [Ca^2+^]_i_ whilst using a fixed depolarisation voltage showed that current conductance was dependent on intracellular calcium (EC_50_ = 227 ± 43 nM), was absent when [Ca^2+^]_i_ was 0 nM and was inhibited at all calcium concentrations by 10 µM Ani9 (Fig. 4b). Conversely, varying the voltage of the depolarising step between − 90 and + 90 mV whilst maintaining intracellular free [Ca^2+^]_i_ at 338 nM showed activated current to be strongly outwardly-rectifying, with the reversal potential coinciding with the calculated chloride equilibrium potential of -20 mV (Fig. 4c). The calcium and voltage-dependency of this chloride-mediated current match the characteristics described for human TMEM16A and differ significantly from any background chloride conductance observed in sham HEK-293 transfection (Supplementary Figure S1).

### *X. tropicalis* Tmem16a has a comparable pharmacological profile to human TMEM16A

We have shown that, like human TMEM16A, *X*. *tropicalis* Tmem16a currents were sensitive to inhibition by Ani9. We extended our analysis of sensitivity to Ani9. Under conditions of maximal conductance (338 nM free [Ca^2+^]_i_ combined with steady-state depolarisation to + 70 mV), the stepwise extracellular application of increasing concentrations of Ani9 caused a concentration-dependent inhibition of *X*. *tropicalis* Tmem16a current (Fig. 4d). The response of *X*. *tropicalis* Tmem16a to Ani9 was identical to that of human TMEM16A in terms of potency (Table 1), with current being blocked over a range of voltages (Fig. 4e). Ani9 inhibited both inward and outward chloride flux, with IC_50_ values at + 70 mV of 0.098 ± 0.009 µM and at - 70 mV of 0.066 ± 0.012 µM (*n* = 13; Fig. 4f, normalised for comparison of large currents at + 70 mV vs. very small currents at - 70 mV).

**Table 1:** Pharmacological profile comparison of *X*. *tropicalis* Tmem16a with published data from human TMEM16A (abc and acd isoforms).

Compound	IC_50_ (*n*) X. tropicalis Tmem16a	Published human TMEM16A whole-cell patch clamp comparisons
Ani9	0.098 ± 0.009 µM (13)	0.066 µM, human TMEM16Aacd^61^ 52% inhibition at 0.05 µM, human TMEM16Aabc^58^ 0.068 µM, human TMEM16Aabc^65^
CaCCinh_A01_	12.91 ± 1.96 µM (9)	7.84 µM, human TMEM16A^62^ 7.57 µM, human TMEM16Aacd^63^ 1.7 µM, human TMEM16Aacd^60^
Benzbromarone	3.30 ± 1.45 µM (10)	3.05 µM, human TMEM16Aabc^65^ 4.09 µM human TMEM16Aacd^63^ 2.35 µM, human TMEM16Aacd^64^
Niflumic acid	85.12 ± 19.06 µM (4)	8.34 µM, human TMEM16Aacd^61^ 12.1 µM, human TMEM16Aacd^60^ 8.54 µM human TMEM16Aacd^63^
CFTR Inh-172	> 30 µM (5)	
Idebenone	> 30 µM (4)	54% and 90% inhibition by 10 µM and 30 µM respectively, human TMEM16A^61^
MONNA	11.89 ± 3.45 µM (6)	13.60 µM human TMEM16Aacd^63^
T16inh-A01	> 30 µM (6)	> 30 µM human TMEM16Aacd^63^ 1.51 µM, human TMEM16Aacd^60^
9-AC	145.9 ± 65.8 µM (5)	57.7 µM, human TMEM16Aacd^﻿60^

Under the same conditions of maximal conductance, we defined concentration-inhibition relationships for a selection of chloride channel inhibitors known to inhibit human TMEM16A (it is noteworthy that CaCCinhA01 is also an inhibitor of CFTR)^60,61,62,63,64,65^. IC_50_ values for *X*. *tropicalis* Tmem16a are given in Table 1, alongside literature values for human TMEM16A from similar whole-cell patch-clamp studies. Side-by-side comparison of these values shows the compounds are similarly potent (within 3-fold of human TMEM16A values) except for niflumic acid, which was found to be markedly less potent at inhibiting *X*. *tropicalis* Tmem16a. There is some dispute in the literature of the effectiveness of T16inh-A01 at blocking human TMEM16A, which in our assay did not directly inhibit *X*. *tropicalis* Tmem16a channel function.

With these electrophysiology studies, we show that *X*. *tropicalis* Tmem16a exhibits the same functional and pharmacological hallmarks as human TMEM16A in terms of its activation by intracellular calcium, voltage-sensitivity, kinetics and relative sensitivities to commonly-used inhibitor compounds.

### Loss of Tmem16a alters mucin secretion and its macromolecular properties

We have shown that *X*. *tropicalis* Tmem16a is present in the SSCs of tadpole skin, and that it functions as a voltage-sensitive, calcium-activated chloride channel in a comparable way to its human counterpart. We hypothesised that *X*. *tropicalis* Tmem16a, presumably via regulation of ion balance, functions in mucin secretion and/or affects its expansion and re-modelling post-secretion from SSCs. To test this, we used a morpholino oligonucleotide (MO) knockdown strategy to deplete Tmem16a from the tadpole skin.

**Fig. 5:**
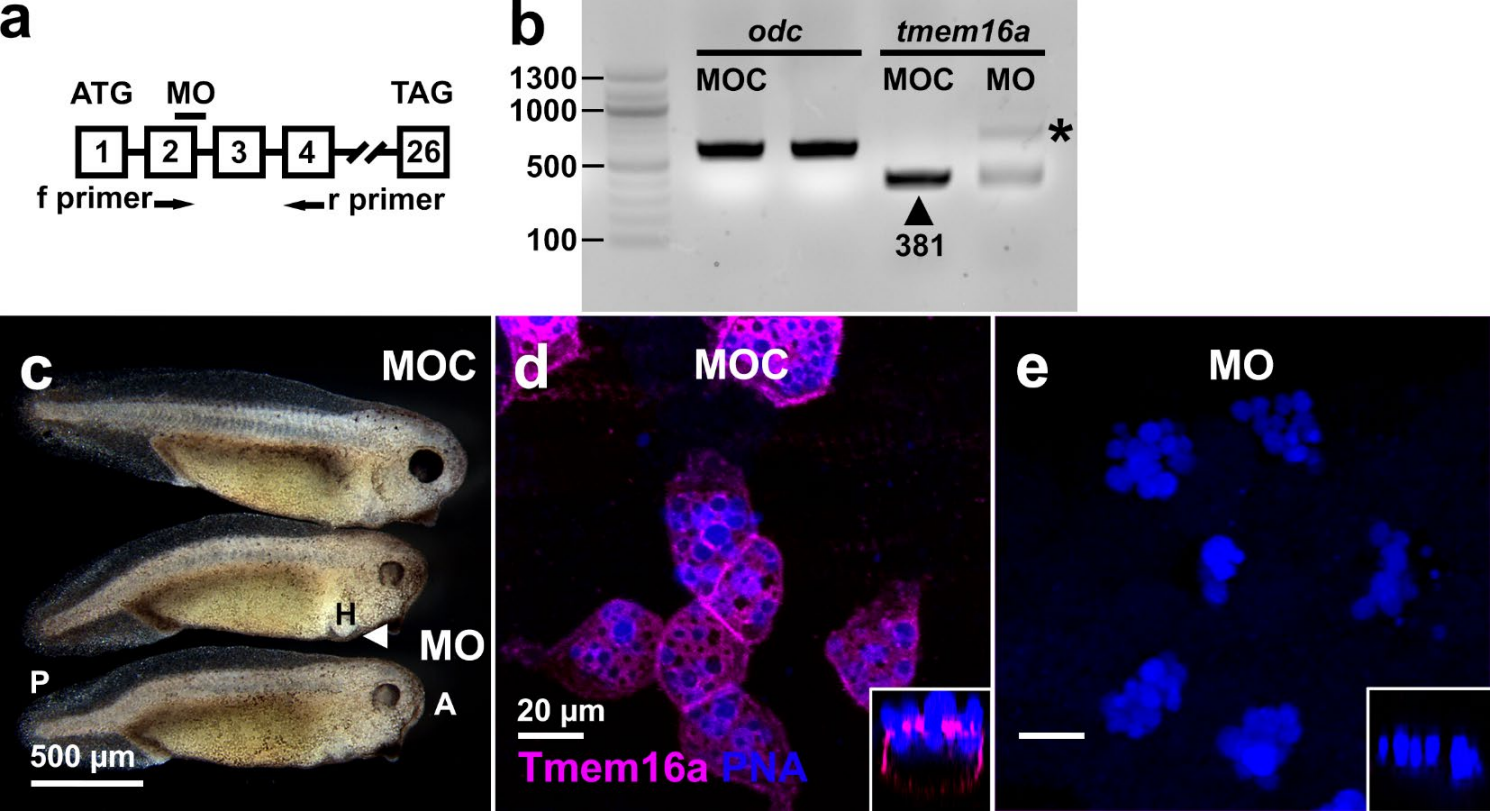
Knockdown of *X. tropicalis* Tmem16a. (**a**) A schematic diagram (not to scale) showing a MO targeted to the splice donor site of exon 2 of *X*. *tropicalis tmem16a* pre-mRNA. Primers used to amplify the resulting mRNA fragment via RT-PCR species are labelled f and r. An arrow indicates the position of a premature termination codon (PTC) in intron 2. Start (ATG) and termination (TAG) codons are indicated in exons 1 and 26, respectively. (**b**) RT-PCR analysis of *tmem16a* mRNA in embryos injected with MOC and *tmem16a* splice MO demonstrate a marked reduction of normally-spliced *tmem16a* mRNA (arrowhead; expected size 381 base pairs in length) in the latter, despite equal loading (amplification of the housekeeping mRNA *odc* was equivalent in both samples). A larger mRNA species resulting from disrupted splicing is evident in MO-injected tadpoles (asterisk). No other amplicons were detected. Fragment sizes were compared against a standard DNA ladder (left lane; sizes indicated in base pairs). (**c**) Morphant embryos have mild anterior-posterior (A, P) defects, delayed head development and small heart (H) edemas (arrowhead). (**d**–**e**) Injection of *tmem16a* splice MO caused a complete loss of Tmem16a protein in the plasma membrane of SSCs in the tadpole skin, marked by PNA staining of the vesicles. Orthogonal views (insets) demonstrate this loss at all cellular planes. PNA staining indicates that SSC development is typical in morphants.

We targeted the donor splice site of exon 2 in *tmem16a* pre-mRNA (Fig. 5a), predicting that full or partial intron retention would result in a premature termination codon after 21 amino acids (Ensembl ENSXETG00000001994)^66^. We tested for disrupted pre-mRNA splicing by RT-PCR across the *tmem16a* target site in cDNA from NF25 embryos (Fig. 5b). In MO control-injected (MOC) embryos, normal splicing of *tmem16a* pre-mRNA was evident by a single amplicon of the predicted size (Fig. 5b, arrowhead). After injection of 15 ng *tmem16a* splice MO into the fertilised egg, we observed a large decrease in signal intensity for the band corresponding to normal splicing of *tmem16a* pre-mRNA and concomitant appearance of a longer amplicon (abnormal splicing; Fig. 5b, asterisk) that likely corresponds to intronic retention and inclusion of a premature termination codon shortly downstream of exon 2. There was no difference in RT-PCR amplification of the housekeeping mRNA ornithine decarboxylase (*odc*), indicating equivalence of samples.

Tmem16a-depleted morphant embryos developed normally until tailbud stages. At NF38, morphant tadpoles displayed a bent anterior-posterior axis, delayed head development, and small edemas around the developing heart (Fig. 5c). However, the morphant tadpoles had a superficially healthy epidermis, and we predicted the observed developmental defects would not impede further investigation. To simultaneously confirm depletion of Tmem16 protein and examine the development of the SSCs in the skin, we performed wholemount immunofluorescence for Tmem16a on MOC-injected and morphant tadpoles at NF38 (Fig. 5d-e). Tmem16a was evident in the plasma membrane of PNA-stained SSCs in MOC-injected tadpoles (Fig. 5d) and this signal was completely lost in morphant tadpoles (Fig. 5e). Orthogonal views (Fig. 5d-e, insets) demonstrated that Tmem16a was not detected at any cellular plane in MO-injected embryos, and that its apparent absence is not an artefact of imaging plane. In these morphant tadpoles, PNA staining of SSCs indicated no detectable impact on the presence or location of mucin vesicles at the apical membrane in these cells. These data show that loss of Tmem16a in the developing tadpole does not impede the development of SSCs. However, cell counts of PNA-positive SSCs in Tmem16a morphants revealed lower numbers of SSCs in a given field-of-view (Supplementary Figure S2), suggesting that Tmem16a affects the differentiation of appropriate numbers of SSCs in this epidermal layer.

**Fig. 6:**
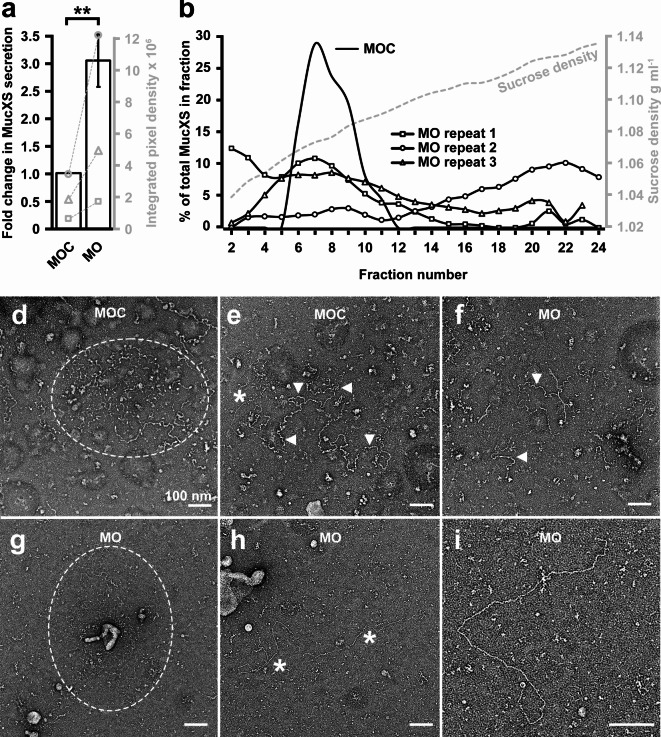
*X. tropicalis* Tmem16a regulates mucin secretion and remodelling. (**a**) Tmem16a depletion results in significantly increased MucXS secretion (mean 3-fold increase) from the tadpole skin under ionomycin-induced conditions. Paired data from three biological replicates is shown on the secondary axis (grey). The Shapiro-Wilk test did not show a significant departure from normality for raw data from either MOC- or MO-injected embryos. Ratio paired t-testing identified a significant difference in MucXS signal between MOC- and MO-injected embryos, *p* = 0.0062. (**b**) MucXS secreted from MOC-injected tadpoles forms a discrete density peak (MOC; typical experiment) while MucXS secreted from Tmem16a morphant tadpoles is distributed across a wide density profile (MO repeats 1–3). Sucrose density from a typical experiment is indicated on the secondary axis (grey). (**d**–**e**) Mucins secreted from MOC-injected tadpoles are detectable as compact (**d**) or semi-expanded (**e**) forms. (**f**–**i**) Mucins secreted from Tmem16a morphant tadpoles are occasionally evident in semi-expanded form (**f**) or as thin strands aggregating with amorphous strutures (**g**). The majority of secreted mucins appear as linear molecules (**h-i**).

We next determined whether Tmem16a depletion affected mucin secretion and/or its macromolecular properties. First, after ten minute exposure to the secretagogue ionomycin, we detected (via slot blotting and immunostaining with an anti-MucXS antibody) a statistically-significant increase in MucXS in the tadpole media, to levels greater than secreted by MOC-injected embryos (Fig. 6a). Although we found lower numbers of SSCs in Tmem16a morphants, this increased level of MucXS in the media may result from a concomitant expansion of the “goblet cell” lineage (Supplementary Figure S2). Alternatively or additionally, Tmem16a in the epidermis may be required to restrict mucin secretion. To assess the impact of Tmem16a depletion on the macromolecular properties of secreted MucXS, media from batches of ionomycin-exposed tadpoles was subjected to rate zonal centrifugation on sucrose gradients (Fig. 6b). This facilitates mucin separation by size/shape, with more compacted mucins moving further through the gradient than expanded mucins. In MOC-injected tadpoles, MucXS typically appears as a discrete density peak. However, in Tmem16a morphants, MucXS signal was detected across the entirety of the density gradient, with variability between biological replicates. In two of three replicates, MucXS signal was shifted towards the lower density fractions (Fig. 6b, MO repeat 1 and repeat 3). These data indicate that secreted MucXS is altered when *X*. *tropicalis* Tmem16a is depleted, and tends to be smaller and/or less compact than MucXS secreted by control tadpoles, although the potential for more tightly-compacted or larger, aggregated MucXS is evident in the shift towards higher-density fractions in one replicate (Fig. 6b, MO repeat 2).

We then examined the size and morphology of the secreted mucins from Tmem16a morphants by transmission electron microscopy (Fig. 6d-i). Mucins secreted from MOC-treated tadpoles are routinely detected in compact (Fig. 6d, within the oval) or semi-expanded (Fig. 6e, arrowheads) forms, consistent with secreted human mucin morphologies previously described^38,67^. In contrast, mucins secreted by Tmem16a morphants were very rarely detected in compact form and only occasionally detected in semi-expanded form (Fig. 6f). We identified a few examples of compact mucins that were aggregated with dense, amorphous structures (Fig. 6g, within the oval), similar to those described in human cystic fibrosis saliva^68^. Although in a compact form, the mucin strands here are visibly thinner than those observed in compact mucins from MOC-injected tadpoles. Overall, the majority of mucins secreted from Tmem16a morphants appeared as expanded, linear molecules (Fig. 6h, asterisk and 6i). Together, these data suggest that Tmem16a is required to limit secretion of mucins and for post-secretory remodelling of mucin molecules.

## Discussion

The Xenopus tadpole skin resembles the upper human respiratory tract, both in cellular composition and its role in mucus barrier formation, providing an attractive model platform to study the biology of the human airway. In this study, we have used the *X*. *tropicalis* tadpole to investigate the normal function of the calcium-activated chloride channel (CACC) Tmem16a in polymeric gel-forming mucin production. The human homologue, TMEM16A, is of interest as a therapeutic intervention for cystic fibrosis, where it is proposed that modulation of TMEM16A function can mitigate the symptoms caused by loss of CFTR.

Mucus secretion is influenced by CACC activity at the apical membrane of secretory cells in healthy mucosal epithelia, and this relationship can become critically important in airway disease^69^. Here, we have shown that Tmem16a is expressed in the mucus-producing epidermal layer of the tadpole, in the SSCs that, by expression of canonical markers, appear most closely related to the GCs in the human airway. Within SSCs, Tmem16a is present in the apical plasma membrane, appropriate for a role in the regulation of mucin secretion and/or remodelling. We have confirmed the CACC activity of *X*. *tropicalis* Tmem16a, and shown its activity and response to inhibitors is comparable to the human TMEM16A homologue. Whole-cell patch clamp analysis shows that *X*. *tropicalis* Tmem16a CACC activity matches that of human TMEM16A - a slowly activating and deactivating current with clear voltage and intracellular calcium sensitivity. Its pharmacological profile is also comparable with human TMEM16A, with the exception of niflumic acid sensitivity, which is 10-fold less potent at inhibiting *X*. *tropicalis* Tmem16a than its human counterpart. This variation may arise as a result of channel sequence variation impacting on niflumic acid binding. *X*. *tropicalis* Tmem16a shares a 77.6% protein sequence identity with human TMEM16A, but varies in a sequence of four amino acids (GMVK) in the first intracellular loop that is highly conserved in other species (EAVK), including human^70^, and this may introduce variation in CACC activity/inhibitor sensitivity.

The generation of a functional mucus barrier involves several steps - gene activation, mucin production/modification, packaging into vesicles, secretion and mucin remodelling/expansion - and there is conflicting evidence for the precise role of TMEM16A-mediated CACC activity in this process. The basal CACC activity of TMEM16A in homeostasis is disputed, with low expression levels in healthy, unstimulated human bronchial cells^22,25,27^ suggesting little/no baseline role. However, measurement of CACC activity in siRNA-mediated TMEM16A-depleted^25,27^/TMEM16A-blocked^71^ cells and in *Tmem16a*^*−/−*^mice^72^ suggests a detectable (and perhaps essential^34^) role in homeostatic mucus barrier formation in the human airway. Under pro-inflammatory conditions, the evidence for TMEM16a CACC activity in mucin production is more consistent. Upregulation of TMEM16A is observed in GCs, concomitant with increased CACC activity which can be rescued by blocking TMEM16A-specific CACC activity^22,25,27,71^. In mammalian airway models, TMEM16A upregulation is accompanied by an increase of MUC5AC production^22,25,73^, specifically in TMEM16A-expressing GCs^22,25^. In human bronchial cells, ATP-induced loss of GC-specific MUC5AC signal requires TMEM16A CACC (it is assumed that reduced MUC5AC signal in these cells is evidence of increased MUC5AC secretion)^22^. Intriguingly, conditional loss of TMEM16A in airway ciliated cells leads to mucus accumulation in bronchial club cells^34^, suggesting an indirect role for TMEM16A in the secretion of mucus, perhaps via regulation of pro-secretory molecules like ATP from ciliated cells. Finally, there are data evidencing that TMEM16A and MUC5AC upregulation in goblet cells may represent decoupled events that co-emerge within the pro-proliferative environment induced during differentiation of stem-like cells or under pro-inflammatory conditions^35^. Tracheal analysis in *Tmem16a*^*−/−*^mice shows expansion of the secretory cell lineages at the expense of the ciliated cell lineage and cilia flow function^74^, and upregulation of TMEM16A has been found in differentiating stem-like cells^35^. Within a developmental framework, TMEM16A may indirectly affect production of mucins at the tissue level by modulating stem/basal cell fate to alter numbers of secretory cells. This mechanism is supported by our data showing changes in the secretory cell landscape in Tmem16a morphants.

Regarding mucus barrier formation, modulation of TMEM16A activity has been shown to affect airway surface liquid (ASL) height^16,35^, suggesting that TMEM16A contributes to ASL hydration by influencing mucin expansion/remodelling post-secretion. However, there are no existing data that address a role for TMEM16A in the regulation of mucin structure post-secretion, and our study provides the first such analysis. Most studies on TMEM16A and mucin production have assessed intracellular, goblet cell-stored mucin (for example^22,25^). In contrast, we have quantified and visualised secreted, not intracellular, mucin. We find that, compared to control tadpoles, loss of Tmem16a protein causes increased MucXS in the tadpole media upon stimulation with ionomycin, suggesting that, in the tadpole skin, Tmem16a has a yet-undefined role in negatively-controlling mucin secretion under pro-secretion conditions. Although apparently contrasting with previous data showing reduced ASL height when TMEM16A activity is blocked^35^, reduced ASL height reflects hydration of the mucus layer rather than necessarily reflecting the amount of mucins within that layer. Further, the specific analysis and the external surface of the two different epithelial systems may be relevant. We have assessed the amount of mucins coming from the mucus barrier into tadpole media over a period of 10 min. Tadpoles are motile, and moving through this media would mechanically promote mucin release. We also predict that the aquatic environment could, via continuous solubilising conditions, promote mucin loss from the barrier proper, particularly if the barrier structure is compromised by changes that promote shedding of mucin into the media samples.

To explore potential changes in barrier structure, we investigated the post-secretory maturation of mucins by assessing the macromolecular properties of MucXS in tadpole media. Using a combination of sedimentation analysis coupled with TEM, we identified changes in mucin forms after Tmem16a loss that are consistent with impaired MucXS expansion/remodelling post-secretion, comparable to those observed for mucins in cystic fibrosis mucus and in vivo and in vitro under conditions that inhibited Cl^−^and bicarbonate secretion^68^. Specifically, sedimentation analysis revealed a tendency for MucXS secreted from Tmem16a morphants to occupy lower density fractions than that secreted from control tadpoles. By TEM, mucins from Tmem16a morphants had atypical morphology and rarely appeared in the compact form observed routinely in mucins from control tadpoles. Instead, they appeared as thinner strands that were fully-expanded and linear. We consider these expanded, linear mucins the most likely reason for the tendency to shift to low density fractions in our sedimentation analysis. We also speculate that this morphology may be more easily lost from the mucus barrier and underpin the apparent increase in secretion into the media we observe in Tmem16a morphants.

The lack of properly remodelled mucins secreted from tadpoles lacking Tmem16a is a novel observation and adds significantly to our understanding of this function of this CACC. Mechanistically, while we predict that loss of chloride trafficking will affect the ionic homeostasis with subsequent effects on mucus remodelling, the effect of loss of Tmem16a on bicarbonate secretion has important implications for mucin remodelling post-secretion. In mucins secreted from mouse *CftrΔ508* ileal mucosa, bicarbonate added to the extracellular environment permits functional remodelling into a healthy mucus barrier^75^, likely by chelating and sequestering Ca^2+^ from newly-secreted mucins^76^. We hypothesise that, parallel to CFTR, loss of Tmem16a in the tadpole skin interferes with bicarbonate movement and subsequent loss of Ca^2+^ chelation and sequestration from the newly-secreted mucin, potentially interfering with its normal expansion. However, this would predict a morphology of condensed mucin aggregates and, while we occasionally observe this form in mucins secreted from Tmem16a morphants, these are rare. The dominant morphology of expanded, linear mucin strands points to a more complex role for Tmem16a in the tadpole skin and/or a differing compensatory CACC network compared to mammalian mucosal epithelial surfaces. In support, the *X*. *tropicalis* tadpole skin does not appear to express the *cftr* homologue in any cell type, although we do detect expression in other structures like the notochord (Supplementary Figure S3). Further, transcription of *cftr* was not evident in the *X*. *tropicalis* single-cell sequencing database^43^ although transcripts corresponding to the TMEM16A regulatory protein CLCA1^77,78,79^ were detected in the SSC lineage. That is, CACC activity in mucus secretion and expansion in the tadpole skin overlaps with but does not fully replicate that of mammalian model systems. TMEM16A and CFTR CACC are functionally interlinked^80,81^ and the tadpole skin would potentially be unable to compensate for Tmem16a loss in the same way as other model systems might. However, a Cftr-null system may prove advantageous for further research on Tmem16a function (including that of regulatory proteins) necessarily-decoupled from Cftr activity. We note that the lack of Cftr in the *X*. *tropicalis* tadpole skin presents an ideal testbed for further understanding of the interaction of Tmem16a with mutated Cftr protein overexpressed in the Cftr-null epithelia.

We have previously demonstrated the utility of the *X*. *tropicalis* skin surface as an in vivo model to interrogate mucin and mucus biology, showing that the gel-forming mucin MucXS underpins a host protective barrier that can trap pathogens and promote survival^38^. Here, we further demonstrate that the *X*. *tropicalis* tadpole skin is an accessible alternative to mammalian airway mucosal epithelium for investigating the role of TMEM16A in the generation of the mucus barrier. In summary, *X*. *tropicalis* Tmem16a functions as a voltage-sensitive, calcium-activated chloride in a comparable way to its human GC-located counterpart. We have confirmed that Tmem16a regulates the typical properties of the mucus barrier by studying mucin secretion in tadpoles lacking Tmem16a, where mucin secretion is elevated above that in control embryos, and those secreted mucins lack the characteristic density and morphological properties found with control embryos.

## Methods

### Single-cell RNA sequencing database analysis

The developmental time series of single-cell transcriptomes in *X*. *tropicalis* embryos^43^ can be found here: https://kleintools.hms.harvard.edu/tools/currentDatasetsList_xenopus_v2.html. Searches for expression of specific genes were performed using the available platform tools. The small secretory cell lineage was identified by marker gene expression within the *all stages* SPRING plot. In *tree view*, relative expression levels across the entire dataset are represented on the platform by variable green luminosity; these relative expression levels in target cell lineages over developmental time were captured from the platform display via the Photoshop (Adobe) colour picking tool. Gene expression clusters of small secretory and goblet cell lineages at Nieuwkoop and Faber (NF) stage 14 were subplotted from the *stage 14* dataset using the default parameters provided by the platform. An outlying cell, assigned by marker expression to the goblet cell lineage, was discarded. For each analysis, scale bars show relative and not absolute expression levels within the dataset, where 0 indicates no/undetectable expression and 1 indicates the maxima for the selected RNA. Thus, green luminosity cannot be used to compare expression levels between different RNAs.

### Animal husbandry, obtaining embryos and fixation/storage

To obtain *Xenopus tropicalis* embryos, adult male and female frogs were primed with 15 units of pregnant mare serum gonadotrophin (PMSG; MSD Animal Health), 18–24 h prior to ovulation. Mating was subsequently induced with 50 units of human chorionic gonadotrophin (HCG; MSD Animal Health) in males and 75 units in females. Hormone injection in adults was performed under United Kingdom Home Office animal project licence numbers PFDA14F2D and PP1859264, were performed in accordance with the relevant protocols within those project licences, by trained personal licence holders. All data presented in this study was obtained from pre-feeding stage (approximately 3–4 days of development from fertilisation) embryos which are not considered protected animals for regulated procedures under the Animals (Scientific Procedures) Act 1986. All experiments using *Xenopus tropicalis* animals are reported according to applicable ARRIVE guidelines for this species. Embryos were maintained in 0.01 X Marc’s modified Ringer’s (MMR) in 1% agarose-coated dishes/multiwell plates at 23–25 °C, and staged according to Nieuwkoop and Faber^82^. At late neurula stages, embryos were removed to uncoated dishes/multiwell plates for the remainder of culture. Unless otherwise indicated in the text, embryos at the required stage were fixed in 1 X MEMFA fixative (100 mM MOPS [pH 7.4], 2 mM EGTA, 1 mM MgSO_4_, 3.7% (v/v) formaldehyde) for one hour at room temperature or 4 °C overnight. Fixed embryos were dehydrated in 100% methanol and stored at -20 °C.

### Embryo microinjection, morpholino oligonucleotides and mRNA overexpression

Embryos for microinjection were de-jellied for approximately five minutes in 0.1 X MMR containing 2% (w/v) L-cysteine (pH 7.8), and washed several times in 0.1 X MMR. Microinjections were performed at NF1/fertilised egg (or, where indicated in the text, NF4/8-cell stage) using a Picospritzer III microinjector (Intracel) to inject 1–4 nl volumes. During injection and for one hour after, embryos were maintained in 0.1 X MMR containing 2% (w/v) Ficoll™ 400 (Thermo Fisher). Antisense morpholino oligonucleotides (MO) and standard control MO (MOC) were designed by and purchased from Gene Tools LLC. MO sequences were: MOC 5′-CCTCTTACCTCAGTTACAATTTATA-3′; *tmem16a* splice MO 5’-AATGTTCATTCTTTTTACCTCTTCA-3’. MOs were reconstituted to 20 ng nl^−1^ in non-DEPC-treated nuclease-free water (Thermo Fisher) and stored in small aliquots in tightly-sealed vials at room temperature. MOs were heated at 65 °C for five minutes then vortexed before dilution/use, to ensure the MO was fully dissolved. *GAP43-GFP* (“membrane-GFP”) mRNA to mark cell membranes was generated by in vitro transcription from a pCS2-*GAP43*-*GFP*plasmid construct^83^. The construct was linearised with NotI, transcribed with SP6 RNA polymerase, and mRNA injected at NF4 in the ventral blastomeres.

### Reverse-transcription (RT) PCR to test MO efficacy

The efficacy of the *tmem16a* MO to disrupt normal splicing of *tmem16a* mRNA was determined by RT-PCR. Total RNA was extracted from pools of five MO-injected, NF25 embryos using the RNeasy Mini Kit (Qiagen) and resuspended in non-DEPC-treated nuclease-free water (Thermo Fisher). 1 µg of RNA was reverse-transcribed using random hexamer oligonucleotides and the High Capacity RNA-to-cDNA kit (Thermo Fisher), according to the manufacturer’s instructions. PCR amplification was performed with primers flanking the *tmem16a* target splice site and primers detecting the ‘housekeeping’ *ornithine decarboxylase* (*odc*). Primer sequences (with expected wild-type product size indicated in brackets) were: *odc* forward primer 5’-GAAAGTGGCAAGGAATCACC-3’ and reverse primer 5’-AAACAAGATGCAGTTGAAAG-3’ (550 bp); *tmem16a* forward primer 5’-ACCATCACAGCAACTCCGTA-3’ and reverse primer 5’-CAACCTTGGGCTGAATTGGT-3’ (381 bp). PCR reactions comprised 1 X GoTaqGreen MasterMix (Promega), 1 µl cDNA reaction and 500 nm each primer, and were performed on a Veriti 96-well fast thermal cycler (Thermo Fisher) using standard PCR methods. PCR products were analysed by agarose gel electrophoresis, visualised with ethidium bromide under UV light, and a digital image of the relevant field-of-view captured (Azure Biosystems c400). Amplicon sizes were compared against a standard DNA ladder (New England Biosciences; 100 bp ladder).

### Whole-mount in situ hybridisation, immunofluorescence and lectin staining

Wholemount chromogenic and fluorescence in situ hybridisation was performed as previously described^84,85^. For in situ hybridisation, plasmid constructs for generating in situ probes were identified and recovered by standard microbiological methods from an *E*. *coli* library of expressed sequence tag (EST) clones^86^. Both *tmem16a* (TTbA054f11) and *spdef* (TTbA011n06) EST clones were linearised with EcoRI and transcribed with T3 RNA polymerase in the presence of digoxygenin-11-UTP (Roche). Immunofluorescence was performed as previously described^87^. For immunofluorescence, rabbit anti-Tmem16a (ab64085; Abcam) was used at a dilution of 1:1000. Where necessary for signal amplification, mouse anti-GFP (ab1218; Abcam) was used at a dilution of 1:500. Fluorophore-conjugated secondary antibodies (Alexa Fluor 488 or Alexa Fluor 568; Thermo Fisher) were used at a dilution of 1:500. For lectin staining, peanut agglutinin (PNA) conjugated to fluorescein (Vector Laboratories) or Alexa Fluor 568 (Thermo Fisher) at a dilution of 1:1000 was added to samples and incubated at room temperature for approximately 30 min or included during secondary antibody incubation. Chromogenic staining and fluorescence were visualised by stereomicroscopy (Leica M165 FC using Leica Application Suite X v5.2.2 software) and fluorescence by confocal imaging (Olympus IX81 using FV10-ASW v4.2 software). The confocal data was 3D surface rendered using IMARIS v10.1 (Bitplane). Each surface was created independently using the fluorescent signal from magenta and blue channels. Surface boundaries were identified using the threshold tool and were adjusted until they tightly fit the peak signal intensities. Individual 3D surface images were combined. Where necessary, images were processed using Photoshop v23.2.1 (Adobe) software to correct for suboptimal dynamic range (Levels tool), reduce background noise (Noise tool) and sharpen appropriately (Unsharp Mask tool). Fiji v2.14.0 software^88^ was used to convert images into colours accesible to colour-blind readers.

### *X. tropicalis tmem16a* expression construct

A pCS2-*tmem16a* expression construct was generated by PCR amplification and cloning of the *X*. *tropicalis tmem16a* coding sequence from cDNA derived from animal cap mRNA (to enrich for epidermal mRNAs). Animal caps were dissected at NF8 and cultured in suspension on agarose-coated dishes in Danilchick’s for Amy (DFA) media until sibling stage NF35. RNA from 10 animal caps was isolated using an RNeasy Mini kit (Qiagen) and reverse transcribed using Superscript IV reverse transcriptase (Thermo Fisher). PCR amplification was performed with primers previously used to clone *X*. *tropicalis tmem16a* from oocytes^89^. Primer sequences were: forward primer 5’-GTACCATTGGTGGTGCGCACAGTATATAG-3’; reverse primer 5’-TCTATCAGTGGAATGAAT GCC-3’. PCR reactions comprised 1 X Phusion High-Fidelity DNA Polymerase (NEB), 1 µl cDNA reaction and 500 nm each primer, and were performed on a Veriti 96-well fast thermal cycler (Thermo Fisher) using standard PCR methods. The PCR product (approximately 3 kb) was isolated via agarose gel electrophoresis and TA-cloned into the pCRII-TOPO vector (Thermo Fisher) and then subcloned into the pCS2 expression vector using BamHI and XhoI sites. The plasmid was amplified using standard microbiological methods.

#### Mammalian cell culture and transfection

HEK Flp-In-293 null cells (Thermo Fisher) were stored, thawed and cultured as described by the supplier, but without antibiotic supplementation of the growth medium. For transfections, cells were seeded in 75 cm^2^ vented culture flasks at a density of 0.018 × 10^6^ cells per cm^2^ and maintained at 37 ^o^C in 5% CO_2_ for 24 h, to ensure approximately 40% confluence at the point of transfection. Cells were transfected with 6 µg of pCS2-*tmem16a* plasmid in a 1:6 volume ratio with transfection reagent GeneJammer (Agilent), according to the manufacturer’s protocol. Transfected cells were maintained in antibiotic-free growth medium at 37 ^o^C in 5% CO_2_ for 24 h. The transfection medium was then aspirated, replaced with complete growth medium containing 1% penicillin-streptomycin (GIBCO), and cells cultured for a further 24 h at 37 ^o^C in 5% CO_2_ to achieve 70–80% confluence prior to electrophysiology studies. HEK cells expressing Tmem16a were harvested using a 2:1 volume ratio of Detachin (AMSBio) and 0.05% Trypsin-EDTA (GIBCO), and resuspended in CHO serum-free medium (GIBCO) supplemented with 25 mM HEPES (GIBCO) and 0.04 mg ml^−1^ trypsin inhibitor (Sigma Aldrich) to a density of 2.5-5.0 × 10^6^ cells ml^−1^. Cells were typically > 95% viable, assessed by trypan blue exclusion using a BioRad cell counter. To prevent clumping of the suspended cells prior to assay, the cell suspension was maintained by stirring gently but continually at room temperature for 20 min to 2 hours.

#### Electrophysiology

Single cell whole-cell patch clamp recordings were performed using the QPatch planar patch-clamp system (Sophion Biosciences). The automated platform resuspended the cells in extracellular buffer containing NMDG-Cl (130 mM), HEPES (10 mM), CaCl_2_ (2 mM) and MgCl_2_ (1 mM), adjusted to pH 7.30 using HCl and 325 mOsm using sucrose. On establishing single-cell giga-Ohm seals, cell membranes were ruptured by suction pulse and dialysed with intracellular solution containing NMDG-Cl (130 mM), EGTA (20 mM), CaCl_2_ (20 mM; calculated to deliver a free intracellular [Ca^2+^] of 338 nM (https://somapp.ucdmc.ucdavis.edu/pharmacology/bers/maxchelator/webmaxc/webmaxcE.htm) at 22 ^o^C to achieve high Tmem16a-mediated current activation for testing inhibitor compounds), HEPES (10 mM), BAPTA (10 mM), MgCl_2_ (1 mM) and Mg-ATP (2 mM), adjusted to pH 7.25 using HCl and 325 mOsm using sucrose. The calcium activation curve was achieved by varying CaCl_2_ concentration from 0 to 25.7 mM to generate a 0-1000 nM range of free intracellular [Ca^2+^].

Whole-cell recordings were performed from a resting membrane potential of - 70 mV, applied across the cell membrane between an intracellular (working) and extracellular (reference) electrode in each QPatch recording well. To activate Tmem16a currents, the membrane was depolarised once every 20 s for a duration of 1 s. For concentration-response experiments, this depolarising step was set to + 70 mV. To test current-voltage relationship, the depolarisations were varied between − 90 and + 90 mV in 20 mV increments. Signals were subject to fourth-order Bessel filtering (sampling frequency 10 kHz, cut-off 3 kHz). Capacitance and series resistance were monitored throughout the recordings; data was only accepted from wells in which these parameters remained stable.

All current data was captured at the end of the 1 s depolarising voltage step. Current time constants (tau_act_ or tau_deact_) were extracted from single phase exponential fit of current activation (from the start of the depolarising step) or deactivation (on repolarisation to - 70 mV immediately after the depolarising step) respectively, using the QPatch software. Tau = 1/C from the fit y = A + B^(−Ct)^, where A is basal current, B is current plateau, and t is time. All replicate data is reported as mean ± standard error of mean (number of replicates).

#### Sucrose density gradient analysis of secreted mucins

Batches of 100 NF42 embryos were collected in 500 µl 0.01 X MMR in a single well of a 24-well plate. To enhance secretion of MucXS, the secretagogue ionomycin (Sigma Aldrich; 1 mM stock in 100% DMSO) was added to a final concentration of 4 µM, and embryos were incubated at room temperature for 10 min with gentle swirling each minute. Media was removed and, where indicated, sedimentation analysis of secreted MucXS performed using rate-zonal centrifugation on sucrose density gradients as described previously^90,91^. 400 µl of media was layered onto 12 ml 10–30% (w/v) sucrose gradients in 14 ml polyallomer tubes (Beckman Coulter), and centrifuged at 151,000 x g for 3 h at 18 ^o^C. Approximately 24 fractions per sample were retrieved by sequential unloading from the top of the tube. MucXS in unfractionated or fractionated media was detected via slot blotting and subsequent immunodetection with an anti-MucXS antibody^38^. Quantification of signal from slot blots was achieved using Fiji v2.14.0 software to capture the raw integrated density of pixels for each signal band. Comparison of signals was performed on raw data using a ratio paired t-test (Prism v10.2.3 software).

#### Electron microscopy analysis

Unfractionated media samples from MOC-injected and Tmem16a morphant embryos induced with ionomycin to secrete MucXS were harvested. 10 µl volumes on Parafilm M were applied to a glow-discharged (25 mA, 30 s) carbon-coated 400 mesh copper grid (Electron Microscopy Sciences) and adsorbed for 1 min. Grids were blotted on Whatman filter paper, then negatively stained with fresh 2% (w/v) uranyl acetate (Agar Scientific) for 1 min, before a final blot and storage at room temperature. Using a Tecnai TALOS 120c operating at 120 Kv (spot 3), data were recorded for 1 s exposures at a nominal magnification of 57,000 × (3.5 Å pixel^−1^). Images were recorded using a CetaS 4 K high resolution camera. Where necessary, images were processed using Photoshop v23.2.1 (Adobe) software to correct for suboptimal dynamic range (Levels tool), reduce background noise (Noise tool) and sharpen appropriately (Unsharp Mask tool).

## Electronic supplementary material

Below is the link to the electronic supplementary material.

Supplementary Material 1

## Data Availability

Data in this manuscript is available upon reasonable request to the corresponding author.
