# Supplementary material for: The Tmem16a chloride channel is required for mucin maturation after secretion from goblet-like cells in the Xenopus tropicalis tadpole skin
Source: Sci Rep. 2024 Oct 26;14:25555. doi: 10.1038/s41598-024-76482-y (PMC11514049; doi:10.1038/s41598-024-76482-y)
Supplement: Supplementary file 1 — Supplementary Material 1 [file 41598_2024_76482_MOESM1_ESM.docx]

**SUPPLEMENTARY DATA**

**S1**

Methods: Pharmacology data was generated from 16 QPatch runs. Transfection efficiency was assessed from the number of whole-cell recordings per QPatch run expressing large (>300 pA), slowly-activating, Ani9-sensitive current. This definition of ‘transfected’ vs ‘untransfected’ was established by comparing transfected cell recordings with ‘sham’ transfects, i.e. cells that had undergone the same transfection process but with the omission of plasmid DNA (Supplementary Figure S1). The ‘sham’ transfects, like untransfected HEK cells, had a small background chloride current generally <300 pA in size (equating to 21.5±3.5 pA/pF (n=14); Figure S1**a**). These currents were rapidly-activating and inactivating, with a time-constant of 16.2±2.4 ms (n=14), showed voltage-sensitivity but were insensitive to block by 10 µM Ani9. Interestingly, they were blocked by 100 µM CaCCinh-A01. In QPatch experiments using transfected cells, approximately 45 % displayed a distinctively large current (1-2 nA or 114.5±25 pA/pF (n=11)) which activated/inactivated slowly with a time-constant of 88.3±8.2 ms (n=11; Figure S1**b**). This current showed outward rectification at positive potentials and sensitivity to block by both 10 µM Ani9 and 100 µM CaCCinh-A01, matching characteristics ascribed to human TMEM16A. Cells with these characteristics were deemed to be expressing *X*. *tropicalis* Tmem16a, and only recordings from successfully-transfected cells which additionally passed seal/series resistance and capacitance quality control criteria, were used in analysis of *X*. *tropicalis* Tmem16a currents.

**a**.
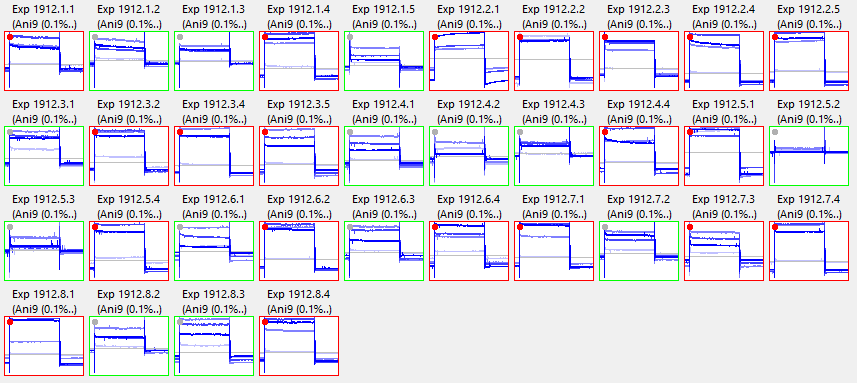


**b**.


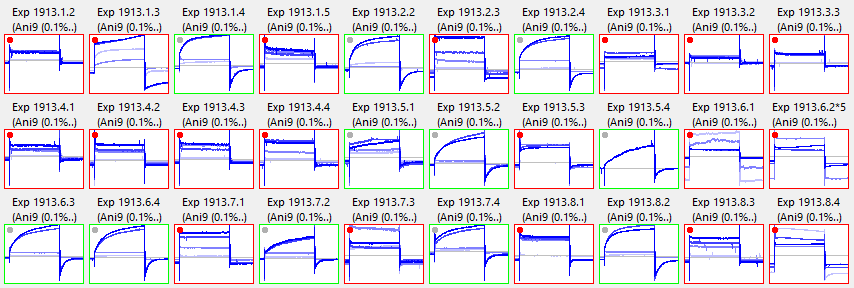


**c**.

**Supplementary Figure S1.** **Identification of successfully-transfected cells**

**a**-**b**. Snapshot of QPatch runs using sham transfects (**a**) and cells transfected with *tmem16a* (**b**). Wells highlighted in green passed quality control assessment and were used in analysis, those in red were rejected (see text for full definitions). **c**. Current levels and Ani9-sensitivity in sham and *tmem16a* transfects which passed quality control assessment.

**S2
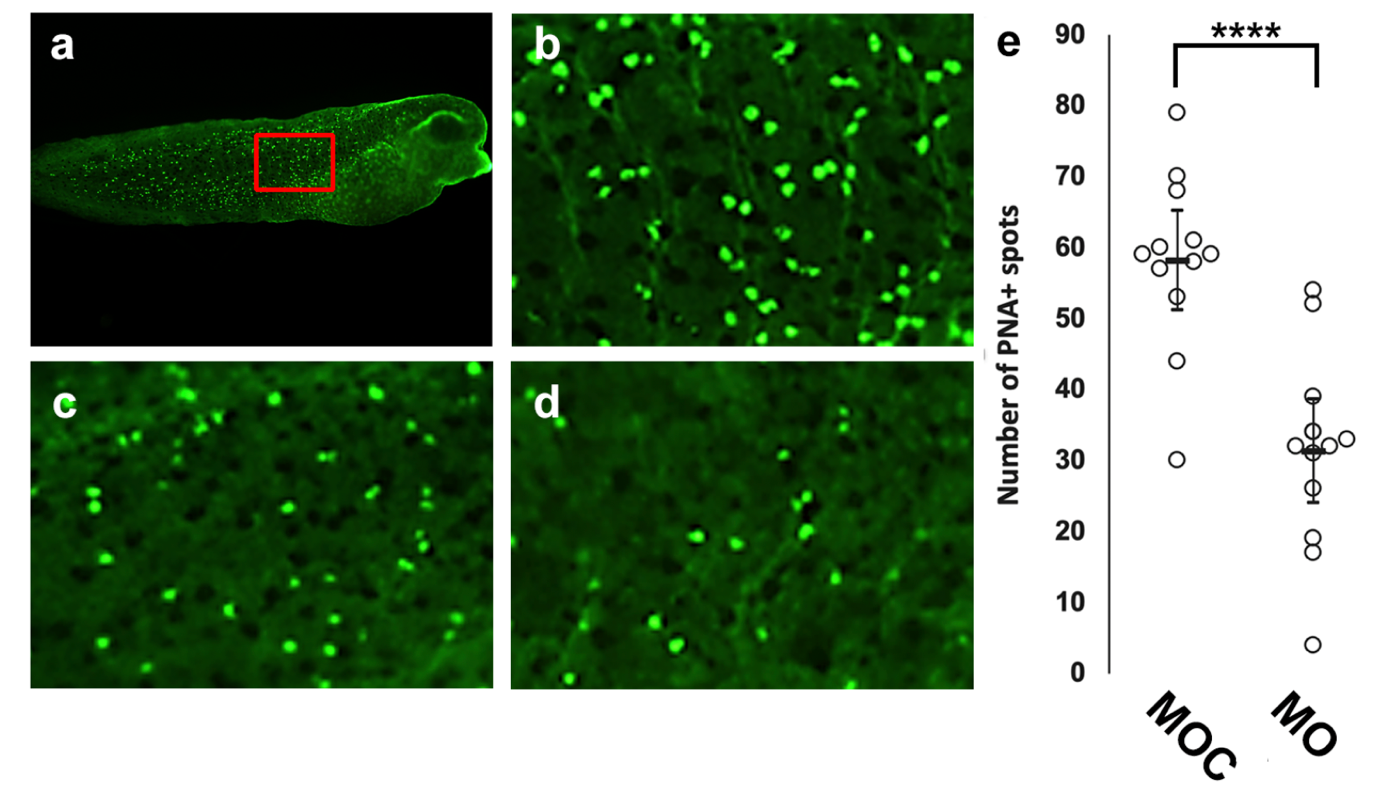
**

**Supplementary Figure S2. Reduced SSC numbers in Tmem16a morphants**

PNA staining was used to detect SSCs in the developing tadpole. PNA-positive cells were counted in a uniformly-sized field of view for MOC and MO-injected embryos (**a**; MOC, red rectangle as an example). MOC embryos had higher numbers of PNA-positive spots per unit area (**b** example) than Tmem16a morphants (**c-d** examples). **e**. Data showing the number of PNA-positive (PNA+) goblet cells in MOC and morphant tadpoles. Student’s t-test revealed the difference to be statistically-significant.

**S3**

**
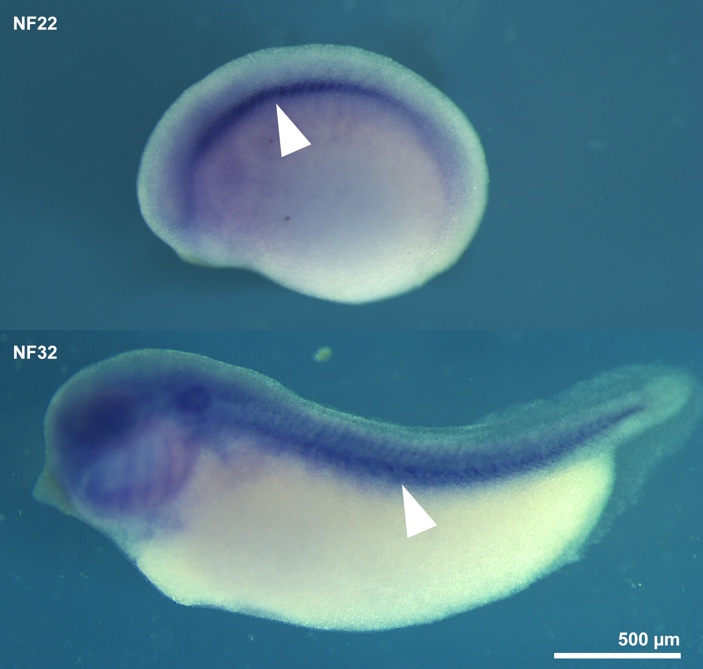
**

**Supplementary Figure S3. Expression of *cftr* at NF22 (upper) and NF32 (lower)**

*In situ* hybridisation to detect expression of *cftr* in the developing tadpole. Expression was not detected in the skin. However, arrowheads indicate expression in the notochord.
